# Design, innovation, and rural creative places: Are the arts the cherry on top, or the secret sauce?

**DOI:** 10.1371/journal.pone.0192962

**Published:** 2018-02-28

**Authors:** Timothy R. Wojan, Bonnie Nichols

**Affiliations:** 1 Rural Economy Branch, Economic Research Service, U.S. Department of Agriculture, Washington, District of Columbia, United States of America; 2 Office of Research and Analysis, National Endowment for the Arts, Washington, District of Columbia, United States of America; University of Georgia, UNITED STATES

## Abstract

**Objective:**

Creative class theory explains the positive relationship between the arts and commercial innovation as the mutual attraction of artists and other creative workers by an unobserved creative milieu. This study explores alternative theories for rural settings, by analyzing establishment-level survey data combined with data on the local arts scene. The study identifies the local contextual factors associated with a strong design orientation, and estimates the impact that a strong design orientation has on the local economy.

**Method:**

Data on innovation and design come from a nationally representative sample of establishments in tradable industries. Latent class analysis allows identifying unobserved subpopulations comprised of establishments with different design and innovation orientations. Logistic regression allows estimating the association between an establishment’s design orientation and local contextual factors. A quantile instrumental variable regression allows assessing the robustness of the logistic regression results with respect to endogeneity. An estimate of design orientation at the local level derived from the survey is used to examine variation in economic performance during the period of recovery from the Great Recession (2010–2014).

**Results:**

Three distinct innovation (substantive, nominal, and non-innovators) and design orientations (design-integrated, “design last finish,” and no systematic approach to design) are identified. Innovation- and design-intensive establishments were identified in both rural and urban areas. Rural design-integrated establishments tended to locate in counties with more highly educated workforces and containing at least one performing arts organization. A quantile instrumental variable regression confirmed that the logistic regression result is robust to endogeneity concerns. Finally, rural areas characterized by design-integrated establishments experienced faster growth in wages relative to rural areas characterized by establishments using no systematic approach to design.

## Introduction

“Bohemia and Economic Geography,” published by Richard Florida in 2002 [1], identified a surprising association between the share of artists in the local labor force and indicators of economic dynamism such as high-tech employment and new firm formation. Numerous follow-on studies have reinforced the original findings by addressing potential problems of omitted variable bias [2] and by testing for an ostensible arts-innovation nexus in various countries [3, 4] and settlement types [2]. One rational explanation for the co-location of artists or arts organizations and more dynamic businesses is an unobserved “creative milieu” attractive to both. In this way, the arts can be thought of as a reliable indicator of creative milieu that attracts workers in creative, non-artistic occupations that the creative class thesis identifies as the true source of economic dynamism. The arts as a direct input to the innovative process is neither necessary nor particularly appealing for framing a sober argument about the contribution of a vibrant arts scene to economic development: the arts help advertise places as tolerant and free-thinking, which are believed to be critical place attributes for where non-artistic talent choose to live.

Evidence of the ostensible arts-innovation nexus is reliant on the critical assumption that greater economic dynamism in an area is the result of heightened levels of innovation at the firm level even though the innovation orientation of firms is never explicitly examined. The failure to identify an association between the arts and the innovation orientation of local businesses opens up the possibility that the existing evidence of an unobserved creative milieu may be spurious. “The arts as the cherry on top” construct provides a plausible, simple explanation for the observed co-location: the economic dynamism of an area may result in more robust support for the arts. In this view, arts organizations and workers—even with their presumed openness to novelty—are not fellow travelers with innovation but merely by-products of it [5, 6].

The objective of this paper is to examine the ostensible arts-innovation nexus at the establishment level, focusing on the simpler contextual environment of rural areas [2]. We estimate both the innovation and design orientation of establishments using a nationally representative sample and identify a strong association between the presence of performing arts organizations and a strong commitment to design. The association is examined by controlling for other community attributes to assess its robustness. This more direct association between the arts in the locale and the design and innovation orientation of individual businesses suggests a more direct connection between the arts, design, and innovation.

Recent experimental data combined with rich qualitative data on the core skill of innovators lend credence to a causal explanation that has largely been eschewed in the economic geography literature: namely, that the arts may play a role in making non-artistic creative workers more innovative. Despite the claim that “[a]ssociating, or the ability to successfully connect seemingly unrelated questions, problems, or ideas from different fields, is central to the innovator’s DNA,” [7] the authors are silent on how that capacity might be directly developed. Indeed, the whole idea of origination from a creative spark is conspicuously absent in the economics of innovation literature [8, 9].

Still, if associative thinking is the mechanism at work in the arts and in innovation alike, then psychological and not solely economics literature should be consulted. An adjacent realm is cognitive neuroscience: free association, for example, does appear to be at the center of artistic creative processes as documented in functional MRI analysis of improvising jazz musicians. In these studies, regions of the brain associated with self-censoring are turned off and regions associated with self-expression are hyper-activated when musicians improvise [10]. Preliminary research also suggests that arts added to a short–course curriculum delivered to both high school students and engineering professionals to develop innovative capacity can augment participants’ ability to develop non-obvious solutions to problems [11]. A study using the UK Community Innovation Survey found that training in art skills (graphic, design, and multimedia skills) combined with science skills (math and engineering) resulted in higher levels of innovative performance [12]. Although this research is far removed from the arts-innovation discussion within economic geography it does suggest a plausible alternative hypothesis for how the arts may impact innovation.

In addition to contributing to the economic geography debate on the nature of the observed association between arts and innovation, this paper also contributes a new perspective on the plausible functional roles of rural areas in a knowledge- or innovation-based economy.

Despite the footloose character of design-oriented innovation that make its rural occurrence possible, its presence in rural areas is assumed to be highly improbable. Design is commonly regarded as a higher-order function in the modern economy, concentrated in large cities, with lower ordered functions such as extraction, fabrication, and production thought better suited to rural economies [13]. Nevertheless, as the share of value-added dollars from production activities continues to decline within the product value chain, it becomes more important to empirically assess the design capacity of rural firms [14, 15].

Our discussion begins by defining art, innovation, and design, to provide a common understanding of the terms used in the analysis. The empirical association between innovation and design within individual establishments, and the prevalence of strong design and innovation orientations among urban and rural establishments, is then assessed. A richer discussion of the literature identifying or refuting the existence of an arts-innovation nexus precedes discussion of cognitive scientific findings on possible neuro-foundations of this nexus. Empirical analyses assess the location proclivities of design-intensive establishments, the robustness of findings after controlling for endogeneity, and the potential economic impact of design-intensiveness on the local economy. The paper concludes with a discussion of the implications for creative place-making, a strategy that uses arts, design, and cultural assets to improve the livability of communities.

## Defining art, innovation, and design

Arriving at a common understanding of terms for this exercise is essential, given their susceptibility to value-laden definitions shaped by personal tastes and preferences. If art is associated simply with endeavors such as opera, ballet, and the symphony; if innovation is defined narrowly as emerging from science and engineering-based R&D; and if design is believed to be the province of mononymous fashion mavens, then not only are the possible interactions constrained, but the probability of identifying rural occurrences are significantly reduced.

At the other end of the scale, overly inclusive definitions fail to capture the potentially unique contributions of these activities to economic, social, and civic welfare. While it is true that “[t]here is no such thing as an undesigned object,” a useful definition should emphasize what is at the core of good design [16]. Similarly, a definition of innovation should encompass more than mere novelty; and, a definition of art, more than mere expression.

The National Endowment for the Arts’ definition for “art” [17] can be usefully summarized as follows: any generative act intended to communicate richly with others using an established or emerging practice. The three requisite ingredients are the generation of novelty, technique that makes the new ideas accessible, and an audience to receive them.

This definition is inclusive of the widest range of expressive, creative endeavors while excluding the bulk of human activity that is non-expressive, imitative, mundane, inept, or solitary. The process underlying the generation of novelty is not identified, but the requirement that the process be directed to rich communication implies that our imaginative, emotional, and other non-rational resources are tapped. What differentiates the input of these common resources as art is the requirement for some level of technical mastery defined by an established or emerging practice. In its simplest terms, art requires a what (original ideas), a how (technique), and a who (an audience).

A discriminating definition of innovation includes parallel requisite elements: a new idea that is valued by consumers, whose practical utility is realized through various development activities [18]. Often manifest in a very different domain, the elements are nonetheless the same: original ideas, technical mastery, and an audience. And yet, differences between the arts and innovation are stark with respect to where we think the new ideas come from, what purposes the new ideas serve, and which practices or innovation activities (techniques) allow those ideas to be realized. From this perspective, examining art and innovation as parallel processes may be an interesting academic exercise, but any real-world implications would appear unlikely.

Design provides a plausible bridge between the two parallel tracks of art and innovation. A useful concise definition of design is a mediation between people and technology that emphasizes aesthetics [19]. The mediation is both an applied art and a development activity critical to innovation. As an applied art, design taps into many of the same non-rational ways of knowing exploited by art generally. As an innovation activity those non-rational ways of knowing are used to make technology more accessible, relevant, or meaningful, given our shared human experience [16]. In addition, design may feed ideas into the innovation process as new things are discovered through “learning by building” or “learning by drawing”.

However, there are also important ways in which design is different from art or other types of innovation activities. The iterative process of design that may explore a number of divergent ideas in the search for what appeals to consumers—or has the greatest utility for them—contrasts with the more unilateral expression from artist to audience. The differences between design and science- and engineering-based innovation activities come mainly from the artificial nature of all design [20]. Design begins with fabrication and is concerned with things that are man-made. Science and applied science (or engineering) are concerned with scientific truths or laws outside made things. Even if engineering is ultimately concerned with the integrity of a made thing—ensuring that its workings comport with the laws of physics—“good engineering” often can be adjudicated without reference to a specific object. For example, utility patent drawings must convey the workability of an invention but are rarely identical to a patented product introduced to the market. In contrast, “good design” never can be adjudicated without reference to a specific object.

An explicit focus on aesthetics is what differentiates invention and innovation from design, and also provides the link to ways of knowing that are non-rational *ex ante*, thus establishing its affinity for the arts. Aesthetics are always explicit in design. Still, aesthetics are not irrelevant to science, mathematics, or engineering, because truths thought to be independent of human experience are necessarily assessed from a human perspective. Mathematical proofs are sometimes described as elegant; engineering masterpieces that hit upon some new simplifying truth are described as beautiful [21]. Aesthetics are not foreign to science, engineering, and invention, but are most often an outcome of the process of discovery.

In design, aesthetics are an explicit—often central—objective at the outset. Form is an integral part of the process of discovery in design, and can feed directly into invention. “Learning by building,” “learning by drawing,” or “learning by using” are the names given to the process of discovery in design, whereby the initial act of creating something new may result in a deeper understanding of the problem itself, leading ultimately to a better solution.

Quantitative evidence suggests that these feedbacks are important. Take the following example. Utility patents provide protection for the way a new invention is used or works, while a design patent provides protection for how an article looks. A little noted asymmetry between utility and design patents is that while 40% of authors of design patents are also authors on utility patents, only 2% of the authors on utility patents are authors on design patents [22]. This suggests that the design process may result in discoveries contributing to new inventions.

## Empirical studies of design’s role in innovation

The role of design in innovation has been vigorously examined by the design community [19, 21], has received some notice in the literature on business strategy [16], but has been largely disregarded in the economics of innovation literature [23, 24]. Similar variation surrounds the role of design in national innovation policy, with some countries (Denmark, Finland, and the United Kingdom) making design central to their national innovation strategy, and others (Sweden, Italy and France) having explicit policies for service or industrial design. Meanwhile, most countries, including the U.S. and Germany, lack an explicit design component in innovation policy [25].

Studies examining the relationship between design and innovation in nationally representative random samples have been rare. Design has always been included as an innovation activity in the Oslo Manual—the international standard for collecting information on innovation promulgated by the OECD—but questions about design’s role in innovation were first seriously considered in the 2005 revision of the manual [26]. Increased emphasis on non-technological forms of innovation, particularly marketing innovation, raised the importance of design as an innovation activity that could be quantitatively measured through expenditures, just as R&D expenditures are measured as an input to technological innovation. Unfortunately, attempts to elicit this information have performed poorly in cognitive tests. Qualitative information on a commitment to design and the role of the design process in product development has been easier to elicit with survey questions and has become the basis for incorporating design questions into innovation surveys. The UK and Denmark have taken the lead in adding design questions to their national Community Innovation Surveys.

The 2005 UK Innovation Survey was the first European Union Community Innovation Survey to incorporate questions that would allow an examination of the links between design and innovation [27]. In addition to asking if firms engaged in any formal design function and the expenditures associated with that activity, the survey also asked if firms registered designs or used the complexity of design as a method to protect innovations.

The findings were striking to the extent that design was most often associated with high levels of innovation; i.e., the introduction of novel products in the market. Although novel innovators only made up 16% of the sample, more than half (54%) of these firms engaged in some form of design activity. In manufacturing, 63% of novel innovators did. The findings also identified a complementary relationship between design expenditures and expenditures on other types of innovation activities, such as investments in R&D and marketing.

The Denmark R&D and Innovation Survey 2010 asked explicitly about respondents’ commitment to design and the role played by design in their product development [26]. The “ladder model” of design developed by the Danish Design Centre and incorporated into the 2010 survey by Statistics Denmark posits that the role of design is perceived differently across firms. At the ground level no design management implied no systematic role for design in the firm. The first rung is systematic design management at the project level, where design is conceived as styling, and design serves as a “last finish” before market launch. The second rung extends design as a function to be integrated throughout the development process, though it does not necessarily direct that development. The top rung is held by firms in which design is a central and directing element in their business strategy.

The Danish survey asks firms to self-identify as one of these four types of design orientation. Concerns over the subjective nature of the survey’s questions are mitigated to some extent by the cognitive testing, which found that respondents understood the differences across the rungs and could accurately place their own firm on the ladder. Similar to the UK survey, a minority of enterprises report using design either as a last finish (5% of respondents), as integrated, although not a determining element (12%) or as an integrated, determining element (7%). The majority of enterprises did not indicate a systematic approach to design.

Nevertheless, enterprises that self-identified as design-integrated (whether a determining element or not) were 3 times more likely to introduce a product, process, organizational or marketing innovation, relative to enterprises that self-identified as having no systematic approach to design [26]. Design-integrated enterprises also demonstrated faster rates of employment growth, value added growth, and productivity growth relative to the non-systematic design enterprises.

Despite the inclusion of design as an innovation activity in the first Oslo Manual, the link between design and innovation had not previously been substantively investigated in Community Innovation Surveys administered by EU member countries. The findings from the UK and Denmark surveys are remarkable for the seemingly oversized role design plays in high-level innovation. Whether this relationship holds for U.S. establishments is an empirical question, made more interesting by the much lower profile that design enjoys in U.S. innovation policy debates, relative to either Denmark or the UK.

## Design and innovation in rural and urban establishments in the United States

The OECD study of the Danish innovation survey provides a useful framework for examining the association between design and innovation in U.S establishments. The empirical challenge is deriving reliable measures of both the design and innovation orientation in self-reported surveys. The development of the 2014 ERS Rural Establishment Innovation Survey (REIS), which forms the basis of this analysis, was motivated by the need for reliable comparisons of rural and urban innovation rates. Latent class analysis (LCA) used to identify unobserved subpopulations of non-innovators, nominal innovators, and substantive innovators is adapted to identify unobserved subpopulations on the rungs of the design ladder. Technical details regarding the innovation and design LCAs along with validation tests are provided in S1 Supporting Information. In the interest of brevity only a conceptual summary and main findings from the LCA exercises are provided below.

### Identifying substantive innovators and design-integrated establishments

The strategy developed for REIS assumes that an establishment’s orientation toward—and capacity for—innovation is strongly correlated with a number of auxiliary questions pertaining to behaviors or attitudes that can be elicited with simple questions. The population of establishments is assumed to be comprised of unobserved subpopulations defined as Substantive Innovators, Nominal Innovators, and Non-Innovators. Latent class analysis is used to classify respondents into one of these subpopulations based on responses to the auxiliary questions.

Conceptually, two thresholds are used to delineate these three classes. Nominal Innovators meet the first threshold of having the rudiments of a continuous improvement program. This factor provides a firm with baseline information to know when a change constitutes an improvement—the minimum requirement for meaningful incremental innovation [28]. Non-innovators fail to meet the first threshold of having the rudiments of a continuous improvement program. So even if the firm has an interest in innovation, it would not possess the information needed to differentiate an objective improvement from good luck. Finally, Substantive Innovators meet the first threshold of having rudiments of a continuous improvement program, but they also meet a second threshold of displaying behaviors consistent with more far-ranging innovation beyond incremental innovation.

Although REIS was not developed to explicitly examine the design orientation of establishments, the collection of design-related questions in the survey do permit identifying both the existence of design capacities and the level of commitment to design in surveyed establishments. The design ladder construct from Denmark analyzed in the OECD study can thus be reasonably approximated [26].

The three rungs of the design ladder applied in this analysis are: 1) No Systematic Design, describing establishments that do not indicate use of in-house or contracted design services, or that demonstrate outputs from design work in the form of intellectual property protections; 2) Design Last Finish, comprised of establishments indicating use of in-house or contract design services but using few, if any, intellectual property protections; and 3) Design-Integrated, comprised of establishments indicating use of in-house or contract design services, using several types of intellectual property protection, and which are more likely to borrow funds for intangible investments. The information available in REIS cannot be used to differentiate design-integrated establishments by whether design is a strategic or merely functional objective, but this distinction appeared to make little difference empirically with respect to the association between design and innovation in the OECD analysis [26].

### The intersection of design and innovation

Table 1 provides information on the intersection of the innovation and design orientations of establishments. The results reported are for the entire sample that includes establishments from both metropolitan and nonmetropolitan counties. A large majority of design-integrated establishments are also classified as substantive innovators (80.2%), while design last finish establishments are also most commonly substantive innovators (51.6%). The off-diagonal cell in Table 1 of interest is the percentage of design-integrated establishments that are classified as non-innovators (9.97%). Before delving more deeply into characteristics of establishments in these seemingly incongruent cells, it is important to examine whether the intersection of design and innovation identified for the entire sample obtains for the nonmetropolitan subsample.

**Table 1:** Innovation orientation by design orientation.

	Design Integrated	Design Last Finish	No Systematic Design	Percent of All Establishments
Percent of All Establishments	8.11%	31.38%	60.50%	
	(0.64%)	(1.01%)	(1.08%)	
Substantive Innovator	80.19%	51.58%	11.5%	30.12%
	(2.87%)	(1.87%)	(0.98%)	(1.04%)
Nominal Innovator	9.83%	31.55%	36.27%	33.09%
	(2.01%)	(1.74%)	(1.45%)	(1.06%)
Non-Innovator	9.97%	16.87%	52.21%	36.79%
	(2.17%)	(1.27%)	(1.49%)	(1.09%)

Source: 2014 Rural Establishment Innovation Survey.

Table 2 provides information on the intersection of the innovation and design orientations in nonmetro establishments. The most critical row is the top one, which confirms that the rural share of design-integrated and design-last-finish establishments is lower than the national average. However, the differences are clearly in degree—the results do not support the presumption that systematic design is absent from rural economies. The intersection of design orientation and innovation orientation for nonmetro establishments resembles what is found in the nation as a whole: design-integrated establishments are overwhelmingly substantive innovators, while a plurality of design-last-finish establishments are also substantive innovators. Here, too, the off-diagonal cells (design-integrated non-innovators and substantive innovators with no systematic design approach) are not empty.

**Table 2:** Innovation orientation by design orientation for nonmetropolitan establishments.

	Design Integrated	Design Last Finish	No Systematic Design	Percent of All Establishments
Percent of All Establishments	4.76%	28.28%	66.97%	
	(0.64%)	(1.01%)	(1.08%)	
Substantive Innovator	83.49%	41.31%	9.07%	22.56
	(5.92%)	(1.63%)	(0.77%)	(0.59)
Nominal Innovator	11.04%	38.09%	41.00%	38.52
	(4.16%)	(1.59%)	(2.49%)	(0.71)
Non-Innovator	5.47%	20.53%	49.93%	38.92
	(2.16%)	(1.35%)	(2.38%)	(0.70)

Source: 2014 Rural Establishment Innovation Survey.

Nonmetro industries that had the highest share of establishments with no systematic design include: Transportation (85.53%), Mining (74.71%), Finance (74.43%), Wholesale Trade (71.61%), Professional Services (69.09%), and Management of Businesses (or Headquarters 68.91%). Comparably lower shares of firms reporting no systematic approach to design existed in the following industries: Performing Arts and Museums (39.48% had no systematic approach to design); Food and Fiber Manufacturing (45.07%), Durable Manufacturing (47.30%), Information (55.04%), and Nondurable Manufacturing (57.79%). Rural manufacturing appears to demonstrate the strongest commitment to design, as the percentage of design-integrated establishments were highest in Food and Fiber Manufacturing (9.88%), Durable Manufacturing (6.22%) and Nondurable Manufacturing (5.53%). It is notable that Food and Fiber Manufacturing is concentrated in rural areas.

The relatively high share of design-integrated establishments in the Food and Fiber sector provides a plausible explanation for the seemingly odd phenomenon of a strong commitment to design in firms not traditionally classified as innovators. An earlier report demonstrated that these traditional rural industries, characterized by low levels of innovation using patent or R&D expenditure measures, have a relatively high share of substantive innovators [29]. Instead, the majority of design-integrated establishments that are classified as noninnovators come from either Wholesale Trade, Information, or Durable Manufacturing. Wholesale Trade establishments may include label design and other sales promotion services for customers while some Durable Manufacturing establishments may be craft-based such as Furniture. The majority of establishments that combine substantive innovation with no systematic approach to design are found in Wholesale Trade, Professional Services, and Finance.

## The association between local attributes and the design orientation of businesses

The identification of a small share of design-integrated rural establishments—and the more common use of design as last finish in rural areas—opens up the question of whether these businesses are randomly distributed or tend to locate in rural places with particular attributes. Because design-integrated establishments are also most likely to be substantive innovators the literature on the assumed nonrandom location of innovative businesses provides a natural starting point for examining how context might be related to design.

The two dominant ways of investigating regional variation in the rate of innovation can be described as an industry/institutional approach and an individual/amenities approach [2]. In the first instance, a region’s disposition towards innovation is described by its innovative milieu that includes industry clusters, specialized labor employed in a cluster, and research universities and labs. The interaction of cluster firms tackling similar problems provides rich spillovers of information that accelerate innovation relative to a standalone firm. In the second instance, the competitive advantage is believed to come from individuals in creative occupations who are attracted to places that provide opportunities for enriching work life and leisure activities: a creative milieu. Information spillovers are believed to occur across these creative occupations, sometimes working in the same industry and other times coming from disparate industries and occupations [30]. These “spillacrosses” are considered to be an important contributor to radical innovation that often requires the combination of ideas from seemingly unrelated areas. “Third spaces” provided by coffeehouses, music clubs, or amateur team sports are believed to provide the soft infrastructure facilitating spillacrosses.

Both innovative and creative milieus were developed to explain innovation phenomena in densely-packed urban areas. However, the creative milieu construct has also been applied in the rural context to explain faster rates of employment and population growth, and new firm formation [2, 31]. The attributes that attract creative occupations to rural areas are somewhat different—focused on natural amenities and opportunities for outdoor activity—but built amenities and opportunities for interaction remain important.

Considering artists as an “indicator species”—a construct borrowed from conservation biology, whereby an easily observed quantity is used to assess the prevalence of a difficult-to-observe environmental condition—a richer creative milieu might be measured as a surplus of artists beyond what would be normally predicted. Viewed from this perspective, places with a richer creative milieu do better in attracting creative class workers than places characterized by a relatively poor creative milieu. Economic performance also rates better in places with a richer creative milieu [2, 4].

An alternative explanation for the observed association between the arts and innovation is not based on an amenity required to attract workers with creative capabilities. Rather, this explanation treats “the arts” as an indirect input into the creative thought processes underlying radical innovation. In contrast to the microeconomic perspective above, which relies on the interaction of creative workers to produce novel combinations of ideas, this new framework regards the arts as the “secret sauce” for the process of far-ranging innovation—a mechanism of action that might best be detected through the cognitive sciences (including neuro-economics). The specific cognitive process that has been identified as the backbone of innovation is associative thinking—“the ability to successfully connect seemingly unrelated questions, problems, or ideas from different fields” [7]. Despite its perceived centrality to innovation, however, the authors stop short of explaining how to develop this process directly. Instead, development of this non-rational process is assumed to grow with the practice of other, rational “discovery skills” they identify: questioning, observing, experimenting, and networking.

Research using functional Magnetic Resonance Imaging (fMRI), which measures activity in specific regions of the brain in real time, has identified at least one activity that does induce associative thought—jazz improvisation [10]. The study found that the area of the brain involved in self-censoring—the process that would inhibit associative thinking—was deactivated during improvisation.

The authors posit that this same process likely characterizes other artistic endeavors in which the creative processes are not as amenable to real-time brain scans as is improvisation. While we would not expect the *Harvard Business Review* to recommend a crash course in jazz improvisation for business executives wanting to increase their innovative capacity, there is evidence suggesting that artistic avocations are commonly pursued by the most innovative minds and more rarely pursued by less august thinkers [32]. A Nobel Laureate drawn at random would be 12 times more likely to pursue an arts avocation than a random member of the National Academy of Sciences. Relative to the general population, the Nobel Laureate would be nearly 30 times more likely to pursue an arts avocation. A study of Michigan State University Honors College science and technology graduates found that lifelong participation and exposure in the arts and crafts yields the most significant impacts for innovators and entrepreneurs [33]. Recent conceptual arguments for the integration of the arts into innovation include American Association for the Advancement of Science panel discussions [34] and a study of how graduates with arts degrees integrate creativity into non-artistic occupations [35].

The counter-argument to the “arts as secret sauce” is the notion of the “arts as a cherry on top”—a metaphor applicable to both individuals and communities. At either scale, the arts can be seen as a luxury: supremely gifted individuals can pursue the arts as an avocation because, unlike their less prolific colleagues, they are not slaving in the lab until 3 am. By the same token, economically dynamic communities are more likely to generate a surplus needed to support the arts. From this perspective, the arts are inert with respect to innovation, despite the presence of a strong observed association.

An important lesson from earlier work examining the individual-amenity explanation is the need to explain the attributes associated with the location of artists before trying to assess some ostensible contribution of the arts to economic dynamism [2]. For example, identifying an association between the employment share of artists and patents might be uninformative if both artists and patents are strongly associated with a third characteristic, such as the presence of a university. In the current analysis, identifying characteristics that might be related to an arts presence and to the design orientation of establishments is essential to understanding the independent association between the two factors.

The point of departure from previous research is a new focus on performing arts organizations with paid employees, rather than on individuals that identify “Artist” (or “Musician” or “Actor”) as their primary occupation. There are several reasons suggesting that the performing arts organization construct is better suited for assessing the “secret sauce” hypothesis.

Most importantly, the presence of a going concern in the arts assures the production of arts output for the surrounding community. Since the actual exposure to the arts by non-artistic creative talent is central to this hypothesis there must be some venue for the local delivery of the arts. In contrast, there is no guarantee that footloose artists are necessarily producing art for the local community. Second, sampling error for performing arts organization data is zero in contrast to potentially large sampling errors for the presence of artists (derived from the American Community Survey), particularly for small-population counties. Finally, controlling for endogeneity of the presence of performing arts organizations is likely to be more feasible than correcting for endogeneity regarding the employment size of a local arts community. This is because rural performing arts organizations are relatively rare and the distinction between their presence or absence is clear cut. In contrast, many rural counties will support a handful of artists with no obvious threshold defining a strong arts presence. However, arts employment will provide a useful robustness test for the performing-arts organization analysis.

**Fig 1:**
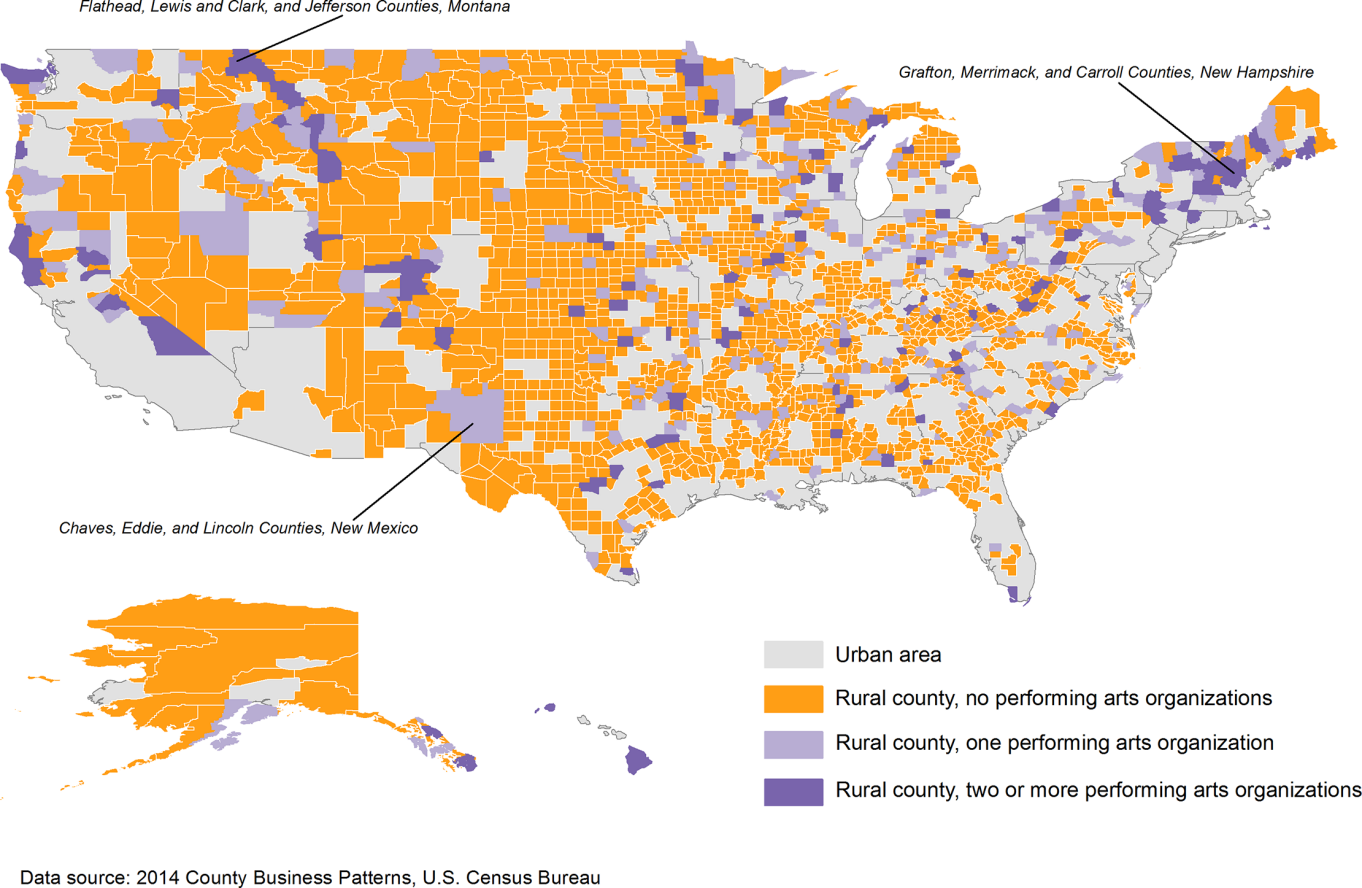
Nonmetro counties with one of more performing arts organizations with paid employees, 2014.

The relative scarcity of rural performing arts organizations with paid employees is displayed in Fig 1, suggesting potential for considerable empirical leverage in explaining the location choices of relatively rare design-oriented business establishments. The map also provides hints of the types of community characteristics associated with such an arts presence. Clusters of counties with more than one performing arts organizations are found in New England, and the Rocky and Sierra Mountains. However, such counties are found throughout the U.S., including in the Midwest, Great Plains, Appalachia, and the South, dispelling the notion that performing arts organizations are found only in high-amenity rural areas that may have little relevance for rural America as a whole.

One of the most distinguishing features of rural areas hosting performing arts organizations is their abundance of natural and recreational amenities. While 34 percent of all U.S. rural counties intersect with a national park or forest, 43 percent of rural areas hosting one or more performing arts organizations are located near parks and forests. Moreover, of rural U.S. counties with performing arts organizations, more than one-third are classified as “recreation-dependent” in a county typology produced by the Economic Research Service [36].

Although few rural counties host performing arts organizations—just 366—these counties are not distributed randomly. Rather, they exhibit geographic clustering. The nearest neighbor ratio among rural counties that host performing arts organizations is 0.515, with an associated p value of <0.001. The ratio is defined as the observed distance between each feature (county with 1 or more performing arts organizations) divided by the expected distance if the each feature was distributed randomly. A ratio below 1 suggests clustering.

Examples of clustered rural counties that host performing arts companies include: Chaves, Lincoln, and Eddy counties in New Mexico (in or near Lincoln National Forest); Flathead, Lewis and Clark, and Jefferson counties in Montana (an area rich in forests and parks near Yellowstone National Park); and Grafton, Merrimack, and Carroll counties in New Hampshire, New York, and Vermont (counties in or around White Mountain and Green Mountain national forests).

The socioeconomic characteristics of rural counties hosting performing arts organization lends some credence to the cherry on top explanation for the ostensible arts-innovation nexus. Residents in these counties are better educated and earn higher incomes than is typical of rural populations. In U.S. rural counties as a whole, for example, the share of adults aged 25 to 44 who have bachelor’s degrees or higher levels of education is 19 percent. In rural counties with two or more performing arts companies, this share rises to 25 percent. The average annual income in rural counties hosting the performing arts is nearly $3,500 higher, on average, than the average income in rural U.S. counties as a whole.

Earlier research has consistently found that the characteristics of multi-unit establishments (e.g., branch plants) are much less likely to be associated with local contextual factors, relative to single-unit firms [37, 38]. Business strategy in multi-unit establishments may be decided in distant corporate headquarters that take little notice of local conditions. Because local conditions are often much more important to the development of business strategy in unit firms, the following analysis is restricted to this subsample of establishments.

**Fig 2:**
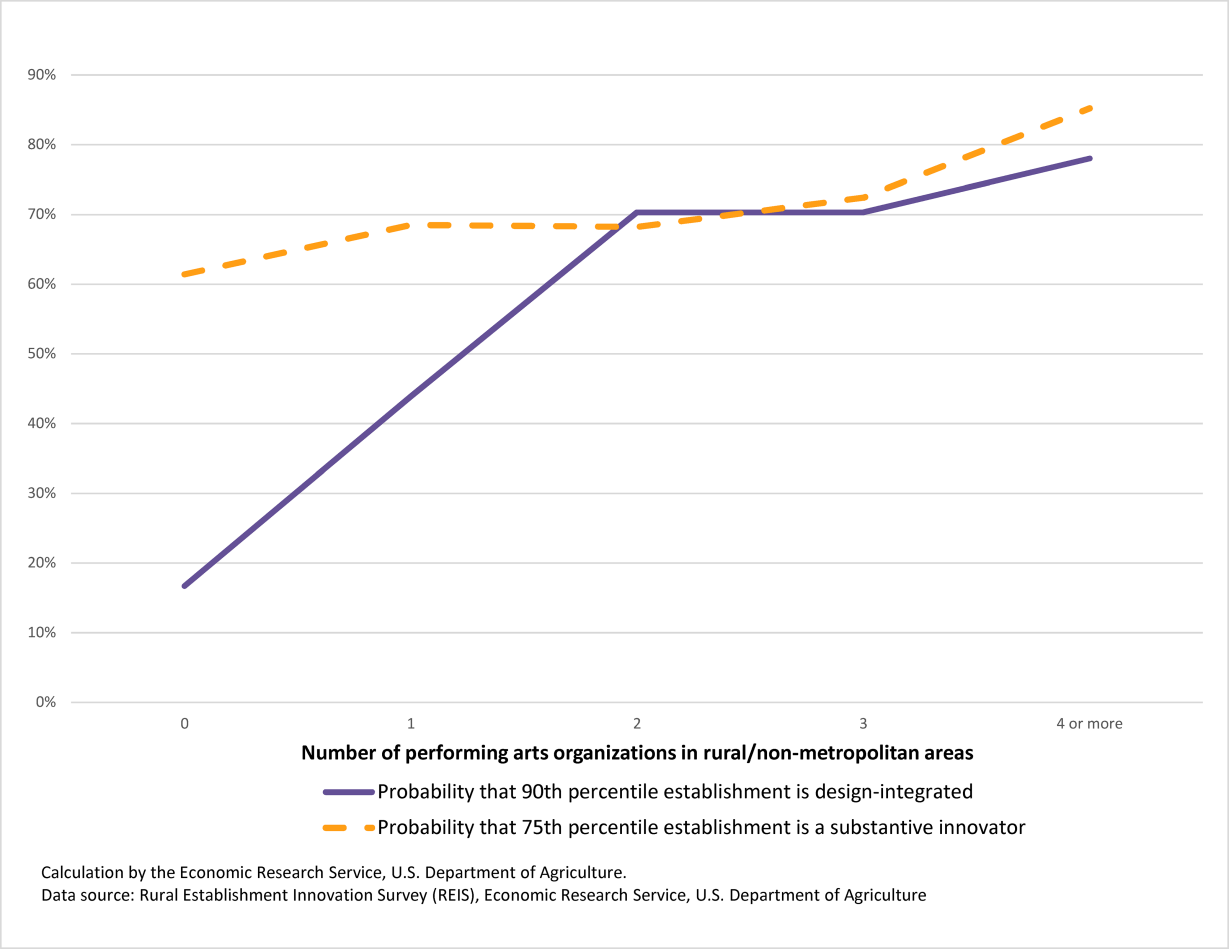
Probability of being classified design integrated or highly innovative varies with number of performing arts organizations in nonmetro county (entrepreneurial establishments).

The business strategy of interest in this analysis is the orientation toward design and innovation. From the latent class analysis discussed above, we have an estimated probability that any establishment is a member of the Design-Integrated, Design Last Finish or No Systematic Design class. Taken together, these probabilities will add to 1 for each establishment, and an establishment is considered to be a member of the class with the highest probability. Since membership in the Design-Integrated class is rare, the most informative statistics are those that examine the right, or uppermost, tail of the distribution. Fig 2 provides a graph of such a statistic—the probability that an establishment is classified as Design-Integrated as a function of the number of performing arts organizations in a county, assessed at the 90^th^ percentile of the Design-Integrated distribution. The majority of rural counties have no performing arts institutions and these same counties have a low probability that the 90^th^-percentile establishment is Design Integrated. However, that probability increases with the addition of performing arts organizations, with the probability of a Design Integrated establishment being high (0.7), with 2 or more such organizations in the county.

The empirical questions suggested by Fig 2 are 1) whether this seemingly strong association is attributable to the existence of arts organizations or is better explained by an omitted variable showing strong associations with both the design orientation and presence of arts organization; and 2) which direction the causality may run, testing whether the existence of design-oriented firms increases the likelihood of a performing arts organization forming. The geographical pattern of rural performing arts organizations, as discussed above, provides important clues on possible omitted variables.

The strong likelihood of performing arts organizations locating near National Parks and National Forests suggest that natural amenities may be an important omitted variable, perhaps affecting design orientation and performing arts organizations differently. The natural amenities and attractions may support the types of tourism flows necessary to sustain performing arts organizations in otherwise sparsely populated rural areas. The amenities input to business strategy would be different, providing an attraction for permanent migrants. This amenity migration may be particularly important for attracting design professionals. Typical of the rural creative class, such professionals often are willing to exchange higher wages from an urban employer for more outdoor recreational opportunities and for a greater quality of life overall [31]. In this case, natural amenities would be the true driver of a design-integrated business strategy, with performing arts organizations being an additional by-product from the rich natural endowment.

Extreme cases of amenity migration, exemplified by places such as Aspen, Colorado or Jackson Hole, Wyoming will also be characterized by very high housing costs. Evidence that design-integrated establishments are more likely to be found in counties with high housing costs would suggest that this business strategy is of limited applicability to most rural places, which are unable to replicate this development model.

The presence of a college offering at least a 4-year degree is another potentially powerful omitted variable. Rural counties with institutions of higher education may be in a better position to support performing arts organizations, given a relatively large population of more highly-educated individuals. The presence of a college may also provide important inputs to establishments with a strong design orientation. Here again, the business strategy and the presence of performing arts organizations might be merely coincidental.

As a statistical method, ordered logistic regression allows examining the association between design orientation and the presence of performing arts organizations while accounting for these alternative explanations. Membership in the No Systematic Design, Design Last Finish, or Design-Integrated class is the phenomenon we hope to explain that is modelled as an ordered logit. The choice of an ordered logit is reinforced by the probability of being classified as Design Integrated rising monotonically through the three ordered classes. Mean probability of being classified as Design Integrated is 0.0008 for the No Systematic Design class, 0.055 for the Design Last Finish Class, and 0.8832 for the Design Integrated Class. The explanatory variables used to explain class membership include the presence of 2 or more arts organizations, a natural amenities scale, the presence of a college granting at least a 4-year degree, the share of the population aged 25–44 with at least a college degree, and the median monthly housing rental cost as explanatory variables. In addition, the establishment size class, the population density of the county, and industry membership (at the 3-digit NAICS level of aggregation) are included as controls.

Results from the ordered logistic regression explaining No Systematic Design, Design Last Finish, or Design-Integrated membership are presented in Table 3. The critical question is whether an independent relationship between performing arts organizations and design orientation still exists after controlling for other community characteristics. All of the coefficient estimates for the explanatory variables are significant. This includes the indicator variable of whether the county has 2 or more performing arts organizations with paid employees confirming that the local arts scene is positively associated with design orientation.

**Table 3:** Ordered logistic regression of design orientation membership as function of establishment and county characteristics.

	Design Integrated		
Variable	Estimate	p-value	Odds Ratio
Intercept DesInt	-2.8670	< .0001	
Intercept LastFin	-0.2488	< .0001	
Establishment Size	0.0165	< .0001	1.017
Population Density	0.0458	< .0001	1.047
4-Year College	0.1775	< .0001	1.194
Share College Grads 25–44	2.8608	< .0001	17.476
Median Contract Rent	-0.00163	< .0001	0.998
Natural Amenity Scale	0.0445	< .0001	1.045
Performing Arts Organizations	0.4136	< .0001	1.512
N	2187		
Percent Concordant	71.1		

Source: 2014 Rural Establishment Innovation Survey. Estimates are qualitatively similar to regression including industry controls.

It is important to note that the Median Contract Rent estimate is negative and statistically significant—an increase in the capacity for and commitment to design does not appear to be associated with pricey, amenity-rich rural counties after controlling for other factors. There appears to be little empirical support for the “cherry on top” explanation of the co-location of performing arts organizations and design-integrated establishments.

The large difference in odds ratios between Performing Arts Organizations and the Share of 25-44-year-olds who are College Graduates is in large part a function of how large a unit change represents. An establishment in a county with arts organizations is roughly 50% more likely to be in the Design-Integrated class. The human capital endowment of the county arguably has a much larger effect on the likelihood of an establishment being Design-Integrated, however. The odds ratio appears enormous (24.357), but this result occurs because a one-unit difference is unlikely ever to be observed (the estimated coefficient represents the difference in odds between a county with no college graduates in the age group and a county where all in the age group have a college degree). More realistically, a county with a college graduate share 10% higher would be 2.5 times more likely to host a Design Integrated establishment.

Artists as a share of the workforce (Florida’s Bohemian measure [1]) was substituted for the Performing Arts Organizations dummy as a robustness check. The results are reported in Table 4. Given that the average share of artists in rural counties is less than 1% (0.74% for the counties with establishments included in the analysis) the estimated coefficient and odds ratio should be interpreted with some caution as was the case with Share College Grads 25–44 above. A coefficient estimate of 31.277 results in an odd ratio that is >999.99. If all workers in a county were artists then design-integrated establishments would be nearly guaranteed. A more reasonable change in the Bohemian Share of 1% still results in roughly a 50% increase in probability of hosting a Design Integrated establishment relative to a Design Last Finish establishment. These findings are qualitatively similar to the Performing Arts Organization dummy, suggesting that the arts generally are positively associated with the design orientation of local businesses.

**Table 4:** Ordered logistic regression of design orientation membership as function of establishment and county characteristics.

	Design Integrated		
Variable	Estimate	p-value	Odds Ratio
Intercept DesInt	-2.9047	< .0001	
Intercept LastFin	-0.2841	< .0001	
Establishment Size	0.0170	< .0001	1.017
Population Density	0.0652	< .0001	1.067
4-Year College	0.1727	< .0001	1.188
College Grads 25–44%	2.2069	< .0001	9.088
Median Contract Rent	-0.00173	< .0001	0.998
Natural Amenity Scale	0.0430	< .0001	1.044
Bohemian Share	38.7286	< .0001	>999.999
N	2187		
Percent Concordant	71.1		

Source: 2014 Rural Establishment Innovation Survey. Estimates are qualitatively similar to regression including industry controls.

While the ordered logistic regression analysis confirms that the statistical association between the presence of performing arts organizations and the design orientation of establishments is robust to alternative explanations, association does not establish causation. In a cross-sectional dataset such as REIS, it is impossible to definitively establish causation; the data provide only a snapshot of each establishment. However, it is possible to test for the possibility of reverse causation—that is, the possibility that the design-integrated establishments of themselves provide an explanation for the presence of performing arts organizations.

### Might design-integrated establishments explain the presence of performing arts organizations?

If rural design-integrated establishments are in fact a critical precursor to rural performing arts organizations, then efforts to increase the viability of performing arts organizations in the hopes of increasing the design orientation of local businesses may be inert. One plausible explanation for such reverse causality is that spouses of design professionals in rural establishments may be more likely to start arts organizations, given limited rural employment opportunities. Another plausible explanation is that design-integrated establishments may be more likely to attract employees that might pursue a performing arts start-up opportunity as a second career. Other explanations are possible. The critical empirical concern is whether the observed association is robust after controlling for the possible endogeneity between design-integrated establishments and performing arts organizations.

The commonly accepted practice in economics to control for endogeneity is to find a valid instrumental variable that does a good job of approximating the explanatory variable of interest. A valid instrumental variable will thus be strongly correlated with the explanatory variable of interest and not (or very weakly) correlated with the dependent variable. The instrumental variable is essentially providing a prediction of the explanatory variable of interest. This predicted value of the variable of interest will not contain the very particular information that would be present if the observed association is the result of endogeneity or reverse causality.

Selecting a valid instrumental variable to control for endogeneity is as much an art as it is a science. Assessing the validity of any selection is more straightforward. The instrumental variable selected for this analysis is the formation of new voluntary organizations in a county between 2000 and 2005. Because many performing arts organizations with paid employees start first as voluntary organizations, a higher formation rate in this earlier period should be positively correlated with the variable of interest in 2014. More generally, a larger number of new voluntary organization will also indicate a healthier environment for social entrepreneurship that is more likely to spill over into the arts domain. At the same time, we do not expect voluntary organization formation to be associated with the business strategy of individual establishments regarding their design orientation.

Empirically, these assumptions are borne out. The tetrachoric correlation between dummy treatment variable (= 1 when 2 or more performing arts organizations are present, = 0 otherwise) and a dummy instrumental variable (= 1 when 39 or more voluntary organizations were formed between 2000 and 2005 in the country, = 0 otherwise; the choice of the threshold was determined empirically; the creation of a dichotomous treatment is required by the quantile instrumental variable estimator used) is a very strong 0.7319 (significant at the < .0001 level). The Pearson correlation between the probability of being a design-integrated establishment and the dummy instrumental variable is a very weak 0.0416 (significant at the 0.001 level).

Because design-integrated establishments are rare, making up less than 5% of rural non-farm tradeable establishments, we would expect the association between performing arts organizations and the probability of being a design-integrated establishment to matter only in the upper (or right) tail of the probability distribution. If we regard the presence of two or more performing arts organizations as a treatment, and the probability of being a design-integrated establishment as the outcome, then the coefficient on performing arts organizations instrumented by voluntary organization formation can be interpreted as a treatment effect.

Instead of estimating the average treatment effect, which would be uninformative, we estimate the treatment effect as it pertains to quantiles in the upper half of the distribution [39]. In the estimation of the instrumental variable quantile treatment effect, we also include controls for two-digit NAICS industry, natural amenities, the share of the 25–44 year old population with a college degree, population density, and the presence of a college granting at least a 4-year degree. The treatment effects at the median (0.5), 70^th^ percentile, 90^th^ percentile and 95^th^ percentile are presented in Table 5 below:

**Table 5:** Unconditional quantile treatment effects under endogeneity (voluntary organization formation instrument).

Treatment Effect of Performing Arts Org on Pr(Design Integrated)	Coef.	Std. Err.	z	Pr > |z|	Lower 95% Confidence Interval	Upper 95% Confidence Interval
@ 50^th^ Percentile	0.00203	.00095	2.14	0.032	0.00017	0.00388
@ 70^th^ Percentile	0.00621	.01736	0.36	0.720	-0.02780	0.04024
@ 90^th^ Percentile	0.22648	.08438	2.68	0.007	0.06109	0.39186
@ 95^th^ Percentile	0.41619	.06237	6.67	<0.0001	0.29395	0.53842

Source: 2014 Rural Establishment Innovation Survey, 2009–2013 Pooled ACS, ERS Natural Amenity Scale, National Center for Charitable Statistics Master File

The quantile treatment effect of two or more performing arts organizations on the probability of being classified as a design-integrated establishment increases markedly at the 90^th^ and 95^th^ percentile. The increase in probability at the 95^th^ percentile is quite large (0.41619) as the largest possible effect would be a value of 1. These treatment effects control for possible endogeneity by using the formation of voluntary organizations from 2000 to 2005 as an instrumental variable. The concern that the observed association between the presence of performing arts organizations and design-integrated establishments is due to reverse causality is not supported if voluntary organization formation is accepted as a valid instrument. However, since the study contains only observational, cross-sectional data, is not possible to test whether the opposite causation obtains.

Longitudinal, experimental or qualitative case studies might be able to address the causal effect of the introduction of performing arts organizations on the design orientation of rural establishments. For now, our data are consistent with the theory that performing arts organizations increase local establishments’ commitment to design, at least for those establishments in the right tail of the Design-Integrated distribution. However, these data may also be consistent with alternative interpretations.

## A design commitment may facilitate innovation, but does it help rural economies?

Within individual establishments, the REIS data on self-reported performance indicators (e.g., entered export markets, share of sales made up of new or significantly improved products, etc.) reinforces the argument that a commitment to design may increase the competitiveness of business firms as reported in Table E in S1 Supporting Information. And at the county level, the REIS data confirm that establishments with a high commitment to design tend to locate in those areas, thus supporting a richer creative milieu. Eventually it will be possible to link design orientation to objective performance data of establishments in REIS by linking with administrative data, providing a more definitive answer on the association between design, performance, and local context.

In the interim, we can examine retrospectively whether rural regions that demonstrated a higher level of commitment to design performed better or worse than rural regions whose establishments tended to have no systematic approach to design. The outcome data come from the Quarterly Census of Employment and Wages, which provides the most comprehensive administrative data on employment, average weekly wages and number of establishments at the county level. The geographical unit for analysis is the 2000 Commuting Zone [40], which groups counties together based on the strength of inter-county commuter flows. Aggregating rural counties in this way increases the sample size for each unit of analysis, providing more accurate estimates of design orientation relative to a county-level analysis.

By examining design orientation and outcomes for each commuting zone by 3-digit NAICS industry, we are able to control for industry structure in our estimation of any economic effect of the design orientation of establishments. In addition, a consistent design orientation at the industry level is much more likely than a consistent design orientation over entire commuting zones. For example, any performance advantage to a design-intensive local furniture industry would be difficult to detect if other local industries had little or no commitment to design.

Two main concerns are raised by the commuting zone-level analysis. First, use of 2014 estimates to “explain” economic outcomes over the period of recovery from the Great Recession (from 2010 to 2014) assumes that design orientation at the commuting zone/industry level is stable over time. Second, the analysis may include potentially large sampling errors, given that the design orientation for a local industry will be estimated from a very small number of establishments. Thus, a truly design-intensive local industry may be misrepresented in the data if the luck of the draw randomly selects a couple establishments that have no systematic approach to design, or vice versa. This sampling error should have no effect on the validity of findings because the errors are random and thus do not bias results. The main effect of large sampling errors will be to reduce the power of the test; that is, a design effect may not be detected in these data even if one truly exists. Technical details of the estimation strategy used in this paper can be found in Appendix C of “Innovation in the Rural Nonfarm Economy: Its Effect on Job and Earnings Growth, 2010–2014” [29].

The regression results shown in Table 6 report estimates of the effect of design orientation of local establishments on employment growth, average weekly wage growth, and change in number of establishments from 2010 to 2014. The regression includes all of the variables that were associated with the design orientation of establishments in Table 3 to ensure that effects from these characteristics are not erroneously attributed to design orientation.

**Table 6:** Associations between local estimates of design orientation and employment and wage growth, and change in number of establishments, 2010–2014.

	Employment Growth		Avg Weekly Wages		Change in Establishments	
Variable	Parameter	Pr > |t|	Parameter	Pr > |t|	Parameter	Pr > |t|
	Estimate		Estimate		Estimate	
Intercept	283.02836	0.0570	-114.103	0.0623	0.71054	0.8409
Pr. Design Integrated	6.22278	0.8623	**52.6855**	**0.0004**	0.30663	0.7184
Pr. Design Last Finish	**53.45583**	**0.0519**	-8.50351	0.4526	0.29557	0.6466
Population	0.00013886	0.4665	-0.0003	< .0001	-0.00002	0.0003
Natural Amenity Scale	32.06436	< .0001	-1.47495	0.4773	-0.01459	0.9028
Median Contract Rent	-0.42491	0.0012	0.02535	0.6392	0.00403	0.1959
Share of 25–44 yo College Grads	79.53659	0.6843	56.89083	0.4799	2.73749	0.5493
Adj R-Sq	0.356		0.1911		0.199	
N	2382		2380		2403	

Pr. No Systematic Design excluded category, industry controls are reported in Table H in S1 Supporting Information. Source: 2014 Rural Establishment Innovation Survey, 2009–2013 Pooled ACS, ERS Natural Amenity Scale, BLS Quarterly Census of Employment and Wages 2010 and 2014.

In addition, the regression includes controls for 3-digit NAICS industry codes (coefficient estimates are provided in Table H in S1 Supporting Information). In the employment growth equation, the coefficient estimate for Probability of Design Last Finish and for the Natural Amenity Scale is positive and statistically significant. The Probability of Design Last Finish effect is relative to the omitted Probability of No Systematic Design. In other words, a commuting zone/industry in which local establishments were assured of having a Design Last Finish orientation (probability = 1) would have added an average of 53 more jobs than a commuting zone/industry in which local establishments were assured of having a No Systematic Design orientation between 2010 and 2014.

The 53 jobs can be interpreted as an upper bound because we are unlikely to observe commuting zone/industries where all of the establishments are assured to possess any particular design orientation. The point estimate of the coefficient on Share of 25–44 Year-Olds with a College Degree is of similar magnitude, but is not statistically significant—the estimate is too imprecise to conclude that this variable had any effect on employment growth. Employment growth was faster in areas with high natural amenities.

In the average weekly wage growth regression, the coefficient estimate for Probability of Being a Design-Integrated establishment is positive and statistically significant. This again is an upper bound, but the impact on wage growth is clear—a worker in a commuting zone/industry where all establishments were assured of being design-integrated would have seen weekly earnings increase $52.68 from 2010–2014, relative to a worker in a commuting zone/industry where all establishments were assured of having no systematic approach to design. As in the employment growth equation, the educational attainment (Share of 25–44 College Grads) coefficient point estimate is similar in magnitude to the design coefficient estimate but is too imprecise to conclude there is any effect on wage growth. None of the estimates of interest in the Change in Establishments equation are statistically significant.

Design did appear to be associated with real benefits in the recovery from the Great Recession for those rural regions where businesses take a systematic approach to design.

## Design, innovation and rural creative places: Possibilities and caveats

The strong association between local arts and economic dynamism in rural areas, as identified in earlier literature, has prompted considerable debate. Since correlation does not necessarily indicate causation, the findings support alternative interpretations that the arts and dynamic establishments are being attracted by the same—though unobserved—factor, termed here as creative milieu, or that the arts are a cultural complement to human capital that facilitates innovative thinking.

As a largely observational science, empirical confirmation of economic phenomena are often asserted when a plausible explanation of some phenomenon is shown to be consistent with empirical data. From this perspective, the “cherry-on-top” explanation for the observed arts-innovation nexus is not supported by the data used in this analysis. However, the data are consistent either with the explanation of the arts as an attractive amenity, or as an enabler of innovative thinking. The economic geography literature has primarily considered the former explanation, although recent experimental research and emerging ways of thinking about innovation lend credence to the latter.

The likelihood that the “arts as enabler of innovative thinking” explanation will ever get a toehold in the economics-of-innovation literature is slim, given its theoretical foundation in non-rational thought, which is anathema to conventional economics. The explanation would appear to lack the plausibility criterion at the outset. But if conventional economics is demarcated by the application of rational thought then the emerging discipline of behavioral economics may provide a more welcoming framework. “Behavioral innovation economics”—attuned to the factors that help explain the generation of novelty through associative thinking—may be better able to get a better handle on the outsized role that imagination plays in the creation of the modern world [8, 9]. The focus in this paper on design may provide a more incremental approach to understanding the role of nonrational inputs to the rational pursuit of developing and selling products on the market. “Designers are…a strategic entry into the problem of how economies and cultures connect because it is their job to make real the social, symbolic and aesthetic currents of their time and location” [41].

The one advantage that the “arts-as-enabler-of-innovative-thinking” explanation has over the “arts-as-attractive-creative-class-amenity” explanation is the availability of experimental data supporting the former premise. The generation of nonobvious solutions, which is arguably a requisite of all art, is likely to be aided by the suppression of activity in those parts of the brain that regulate self-censoring. Limb and Braun [10] provide experimental evidence of this process by scanning the brain function of improvising jazz musicians. Given this scientific description of associative thought working in the musician’s mind, the continued mystery regarding associative thought in the innovator’s mind may finally be addressed from a new angle [7]. Recent research linking arts training of scientists and engineers to an increased capacity for finding nonobvious solutions provides initial evidence of a functional role for the arts in an arts-innovation nexus [11, 12]. A limitation of the present research findings is that we can only posit a connection between the arts and the design/innovation processes in respondent establishments rather than directly observing this connection. This factor raises the question of whether the presence of arts organizations in rural creative places is enough to increase the artistic imagination of residents that is manifest in attributes such as a stronger design orientation of local businesses.

The actual mechanism by which the arts enable innovation in local economies may be important for the types of arts policy and programming that have the highest economic development payoff. Clearly, if the arts serve a functional role in promoting more innovative thinking, then increasing arts participation and appreciation may warrant greater attention. Alternatively, if the arts serve mainly to advertise a place as welcoming to nonartistic creative talent, then recruiting the arts and marketing arts districts or events may be the appropriate focus. In either case, the conclusion of this research is that as a local development strategy, promoting the arts is likely to have a positive impact that extends beyond a direct economic impact to affecting the capacities of businesses reliant on design and innovation, either by attracting or enabling creative talent.

## Supporting information

S1 Supporting Information Latent class analysis using Rural Establishment Innovation Survey data, validation tests, and regression results with industry controls. (DOCX)
