# Supplementary material for: Design, innovation, and rural creative places: Are the arts the cherry on top, or the secret sauce?
Source: PLoS One. 2018 Feb 28;13(2):e0192962. doi: 10.1371/journal.pone.0192962 (PMC5831055; doi:10.1371/journal.pone.0192962)
Supplement: S1 Supporting Information — (DOCX) [file pone.0192962.s001.docx]

# Supporting Information S1: Latent class analysis using Rural Establishment Innovation Survey data, validation tests, and regression results with industry controls

Timothy R. Wojan

Economic Research Service/USDA, Washington, DC, USA

E-mail: [twojan@ers.usda](mailto:twojan@ers.usda)

Here I discuss the methods used to derive the classifications of innovation and design orientation used in the principal paper and assess the validity of the classifications with data contained within the Rural Establishment Innovation Survey and external data

## Identifying substantive innovators

Self-reported innovation surveys have been criticized for not being able to differentiate between innovation leading to favorable market outcomes and innovation merely representing newness, which may not be highly valued by consumers. This deficiency of self-reported innovation surveys is particularly problematic in trying to assess the innovative capacity of rural firms. For example, studies using the European Union CIS consistently show high rates of innovation in thin and less technologically advanced markets—places not assumed to be hotbeds of innovation [1]. If there is a tendency for conventional self-reported methodology to inflate innovation estimates in such environments then findings on the innovative capacity of rural regions would likely be inconclusive. For this reason, the development of REIS focused on a strategy for deriving reliable measures of substantive innovation to provide an accurate assessment of the innovative capacity of U.S. rural establishments.

The strategy assumes that an establishment’s orientation toward—and capacity for—innovation is strongly correlated with a number of auxiliary questions pertaining to behaviors or attitudes that can be elicited with simple questions. The population of establishments is assumed to be comprised of unobserved subpopulations defined as Substantive Innovators, Nominal Innovators, and Non-Innovators. Latent class analysis is used to classify respondents into one of these subpopulations based on responses to the auxiliary questions.

Conceptually, two thresholds are used to delineate these three classes. Nominal Innovators meet the first threshold of having the rudiments of a continuous improvement program. This factor provides a firm with baseline information to know when a change constitutes an improvement—the minimum requirement for meaningful incremental innovation [2]. Non-innovators fail to meet the first threshold of having the rudiments of a continuous improvement program. So even if the firm has an interest in innovation, it would not possess the information needed to differentiate an objective improvement from good luck. Finally, Substantive Innovators meet the first threshold of having rudiments of a continuous improvement program, but they also meet a second threshold of displaying behaviors consistent with more far-ranging innovation beyond incremental innovation.

Empirically, questions to establish the first threshold ask whether the firm specifies the training requirements for each employee and tracks completion; how frequently the firm assesses customer satisfaction using various methods; and how frequently the firm fixes problems identified in customer complaints. Respondents are also asked if the firm uses enterprise resource planning software—integrating applications from accounting, logistics, marketing, etc.—that might further either a continuous improvement program or support more far-ranging innovation.

To examine more far ranging innovation, it is assumed that firms engaged in this activity will have unique experiences not shared by nominal or non-innovators. Firms pursuing far-ranging innovation are more likely to recognize the value of failed but productive innovation initiatives and thus are more likely to acknowledge them in a question about aborted innovation projects. Similarly, these firms are more likely to have confronted the serious asymmetric information problems in innovation finance, and thus be more likely to identify capital constraints in this area. Finally, firms pursuing far-ranging innovation are more likely to produce intellectual property worth protecting using various methods that are less arduous but with wider applicability than patents.

Responses to these questions and whether the respondent introduced a new or significantly improved product, service, process, marketing, or logistics innovation in the past three years, are used in a latent class analysis to identify the conceptual subpopulations [3]. The probabilistic parameterization of the model requires two types of categorical variables—observed or manifest indicator variables and unobserved or latent variables—and two type of parameters: latent class and conditional probabilities [4]. The assumption is that the relationship between these manifest variables can be explained by the latent variable—in this case, innovator class membership. For ease of exposition, if we assume our single latent variable (innovator class membership) X is explained by two manifest variables (A and B), then the latent class model can be expressed as the product of the latent class probabilities ($\pi_{t}^{X})$ and conditional probabilities ($\pi_{it}^{A|X}, \pi_{jt}^{B|X}$):

$\pi_{ijt}^{ABX}=\pi_{t}^{X}\pi_{it}^{A|X}\pi_{jt}^{B|X}$ (1)

For this analysis, the latent variable (X) is innovator class membership (1 = substantive innovator, 2 = nominal innovator and 3 = non-innovator). One of the manifest indicator variables (A) is the use of intellectual property protections other than patents or copyright (*i* = 1, used; *i* = 2, not used). The conditional probability $\pi_{11}^{A|X}$ is the probability that a substantive innovator, selected at random, used intellectual property protections. The full LCA model uses the 8 manifest variables described above.

If the latent variable were in fact observed, then the probabilistic parameterization would merely be a logistic regression on categorical independent variables. Since this is not the case, the latent class structure must be found by using an iterative, maximum-likelihood estimation protocol called expectation-maximization. The simplest way to think of this protocol is as a logistic regression with missing data in the dependent variable. The expectation step of the protocol computes the expected value of the log of the likelihood function, conditional on initial parameter estimates and observed data. The maximization step updates values of the parameter value to maximize the likelihood function. The expectation step is then repeated with these updated values. This iterative process continues until a predefined level of precision is reached.

## Identifying Rungs of the Design Ladder

Applying the same approach to easily observed phenomena, to differentiate an unobserved design orientation of establishments, is also possible. The main difference in the two applications is that auxiliary design questions were not expressly developed for this purpose as was the case for the auxiliary innovation questions. However, the collection of design-related questions in the survey do permit identifying both the existence of design capacities and the level of commitment to design in surveyed establishments. The design ladder construct from Denmark analyzed in the OECD study can thus be reasonably approximated [1].

Because design is ubiquitous, it is impossible to define a design orientation as not existing in a business. Behaviors that display an explicit approach to design provide the first threshold to delineate establishments. Behaviors that demonstrate a strong commitment to design provide a second threshold.

The three rungs of the design ladder applied in this analysis are: 1) No Systematic Design, describing establishments that do not indicate use of in-house or contracted design services, and that fail to demonstrate outputs from design work in the form of intellectual property protections; 2) Design Last Finish, comprised of establishments indicating use of in-house or contract design services but using few, if any, intellectual property protections; and 3) Design-Integrated, comprised of establishments indicating use of in-house or contract design services, using several types of intellectual property protection, and which are more likely to borrow funds for intangible investments. The information available in REIS cannot be used to differentiate design-integrated establishments by whether design is a strategic or merely functional objective, but this distinction appeared to make little difference empirically with respect to the association between design and innovation in the OECD analysis [1].

## Questions Used in the Latent Class Analyses

Questions used to identify rudiments of a continuous improvement program in innovation orientation LCA:

Q26. How often are processes changed to fix problems identified through customer complaints?

__ Never ___Occasionally ___ Regularly

 Q25. How often does this business monitor customer satisfaction through analysis of complaints,

customer satisfaction surveys, focus groups, or other methods?

__ Never ___Occasionally ___ Regularly

Q13. Does this business have written position descriptions?

Are training requirements documented in those position descriptions?

Does this business track whether workers complete or if they have

already completed these training requirements?

___ Yes ___ No

Question used to identify data-driven decision-making in innovation orientation LCA:

Q14. Are the following technologies currently used at this business? …

An integrated enterprise resource planning system (e.g., SAP or Microsoft

Dynamics, or Oracle Applications that include accounting, logistics,

human resources, sales management, along with other functions)

___ Yes ___ No

Questions used to identify more far-ranging innovation initiatives in innovation orientation LCA:

Q28 In the past 3 years, did this business have any improvement or innovation activities that were…

Abandoned ___ Yes ___ No Incomplete ___ Yes ___ No

Q37. In the past 3 years, did this business…

Use trade secret protections (e.g., non-disclosure agreements, non-compete clauses, or sought remedies for misappropriation) ___ Yes ___ No

Q34. In the current environment, if excess cash were available, how likely is it that these funds would be used to…

Fund additional innovation projects

___ Not at all likely ___ Probably ___ Most definitely

At least one positive response to following questions used to identify self-reporting innovators in innovation orientation LCA:

Q27. In the past 3 years, did this business…Produce any new or significantly improved goods?

Provide any new or significantly improved services? Introduce new or significantly improved methods of manufacturing or producing goods or services? Introduce new or significantly improved logistics, delivery, or distribution methods for your inputs, goods, or services? Introduce new or significantly improved support activities for your processes? Introduce new or significant improvements in your marketing methods?

Questions used to identify any systematic approach to design in design orientation LCA:

Q33. In the past 3 years, did this business engage in any of the following innovation-related activities? …

Conduct in-house design activities to improve aesthetics of product or packaging
 ___ Yes ___ No

Purchase design services
 ___ Yes ___ No

Questions used to strong commitment to design in design orientation LCA:

Q37. In the past 3 years, did this business…

Register an industrial design ___ Yes ___ No

Register a trademark ___ Yes ___ No

Produce materials eligible for copyright ___ Yes ___ No

Q46. How were the funds that this business borrowed or wanted to borrow to be used? …

Investment in intangible assets, such as branding, training, or design
 ___ Yes ___ No

Question used to increase likelihood that activities in Q33, Q37, and Q46 were being used in support of establishment’s own innovation activities:

Q34. In the current environment, if excess cash were available, how likely is it that these funds would be used to…

Fund additional innovation projects

___ Not at all likely ___ Probably ___ Most definitely

## Innovation and Design Orientations of Rural and Urban Establishments

Table A provides results from the LCA applied to the 2014 REIS data to identify the innovation orientation of establishments. The results reported are for the entire sample that includes establishments from both metropolitan and nonmetropolitan counties. Substantive innovators are identified least frequently, making up 30.1% of tradable, nonfarm establishments with 5 or more employees. Nominal innovators with rudiments of a continuous improvement system make up 33.1% of establishments, while non-innovators make up the largest share at 36.8%. The justification for the names given to these groups is provided by examining those characteristics that typify each business type. The significant majority of establishments classified as substantive innovators report abandoned and/or incomplete innovation projects (72.3%), or report efforts to protect intellectual property (55.8%).

**Table A: Responses to Questions Used to Classify Establishments by Innovation Orientation**

|  | Substantive Innovator | Nominal Innovator | Non-Innovator |
| --- | --- | --- | --- |
| Percent of All Establishments | 30.12% (1.04%) | 33.09% (1.06%) | 36.79% 1.09%) |
| Abandoned and/or Incomplete Innovation | 72.35%* (1.65%) | 16.72% (1.06%) | 22.09% (1.91%) |
| Probably Use Surplus Funds for Innovation | 46.86% (2.16%) | 47.55%* (2.27%) | 30.85% (2.30%) |
| Most Definitely Use Surplus Funds for Innovation | 47.07%* (2.16%) | 16.34% (1.75%) | 17.11% (2.02%) |
| Trade secret protections | 55.79%* (2.08%) | 13.28% (1.26%) | 13.45% (1.43%) |
| Use ERP Software | 52.49%* (2.17%) | 37.12%* (2.09%) | 15.74% (1.59%) |
| Track Employee Training | 58.78%* (2.01%) | 45.48%* (1.99%) | 10.30% (0.82%) |
| Monitor Customer Satisfaction Regularly | 56.19% (2.10%) | 50.88%* (2.07%) | 3.18% (0.24%) |
| Monitor Customer Satisfaction Occasionally | 37.56% (2.07%) | 44.21% (2.05%) | 36.53% (2.12%) |
| Fix Customer Complaint Problems Regularly | 64.33% (1.83%) | 70.89%* (1.93%) | 17.43% (1.14%) |
| Fix Customer Complaint Problems Occasionally | 28.76% (1.83%) | 29.12%* (1.93%) | 66.73% (1.79%) |
| No Reported Innovations | 0.89%* (0.17%) | 30.95%* (1.79%) | 58.01% (1.73%) |

*Source: 2014 Rural Establishment Innovation Survey.*

** indicates estimate is statistically different from estimate to right at 5% level.*

In contrast, a small minority of both non-innovators and nominal innovators answered any of these questions affirmatively. The share of establishments that would “probably” use surplus funds for additional innovation projects was fairly similar across the business types. However, very few substantive innovators would not use surplus funds for innovation projects while the share that would “most definitely” use funds for this purpose was 3 times that of the nominal and non-innovator establishments.

Table B provides results from the LCA applied to the 2014 REIS data to identify the design orientation of establishments. The results reported are for the entire sample that includes establishments from both metropolitan and nonmetropolitan counties. The percentage of establishments classified as design-integrated (8.1%) is substantially lower than the percentage of establishments classified as substantive innovators (30.1%). To the extent that substantive innovation is a phenomenon observed in a minority of establishments, a strong commitment to design appears to be rare. Indeed, the majority of establishments demonstrate no systematic approach to design (60.5%). Consistent with the design-ladder construct, in-house or contracted design services are common in both Design-Integrated and Design Last Finish establishments and exceedingly rare in No Systematic Design establishments. The differentiation between Design Integrated and Design Last Finish is derived from the intellectual property variables, with trademark registration showing the sharpest contrast between the two classes.

Two of the variables used in the design orientation classification that require additional explanation are those related to finance. “Innovation Capital Constrained” is equivalent to the “Most Definitely Use Surplus Funds for Innovation” variable used in the innovation orientation classification. While this inclusion may appear to confound any relationship between design and innovation orientations, the introduction of new or improved goods is in fact a precondition for design. A business producing a uniform commodity would have no use for design. “Innovation Capital Constrained” allows a modest amount of discrimination between innovators and non-innovators but does not necessarily compel a strong association between design-integrated and substantive innovator status. “Borrowed Funds for Intangible Investments” is an indicator of the commitment to design as design is an example of intangible investment included in the survey. Investments in “branding” are another intangible that may demonstrate a strong commitment to design.

**Table B: Responses to Questions Used to Classify Establishments by Design Orientation**

|  | Design  Integrated | Design Last  Finish | No Systematic Design |
| --- | --- | --- | --- |
| Percent of All Establishments | 8.11% (0.64%) | 31.38% (1.01%) | 60.50% (1.08%) |
| In-House Design | 74.92%* (4.01%) | 60.76%* (1.88%) | 0.04% (0.02%) |
| Contract Design Services | 57.64%* (3.87%) | 42.69%* (1.90%) | 0.79% (0.39%) |
| Innovation Capital Constrained | 53.78%* (4.11%) | 32.63%* (1.80%) | 9.00% (0.89%) |
| Registered Industrial Design | 31.61%* (3.51%) | 0.97%* (0.24%) | 0% (0%) |
| Registered a Trademark | 95.28%* (1.48%) | 11.11%* (1.08%) | 0.84% (0.23%) |
| Produced Copyright Eligible Materials | 83.48%* (3.07%) | 17.15%* (1.36%) | 1.26% (0.27%) |
| Borrowed Funds for Intangible Investments | 19.24%* (3.24%) | 8.62%* (1.02%) | 0.14% (0.07%) |

*Source: 2014 Rural Establishment Innovation Survey.*

** indicates estimate is statistically different from estimate to right at 5% level.*

Table C provides information on the intersection of the innovation and design orientations of establishments. The results reported are for the entire sample that includes establishments from both metropolitan and nonmetropolitan counties. A large majority of design-integrated establishments are also classified as substantive innovators (80.2%), while design last finish establishments are also most commonly substantive innovators (51.6%). The off-diagonal cell in Table C of interest is the percentage of design-integrated establishments that are classified as non-innovators (9.97%). The intersection of design and innovation for the nonmetropolitan subsample is examined next.

**Table C: Innovation Orientation by Design Orientation**

|  | Design Integrated | Design Last Finish | No Systematic Design |
| --- | --- | --- | --- |
| Percent of All Establishments | 8.11% | 31.38% | 60.50% |
|  | (0.64%) | (1.01%) | (1.08%) |
| Substantive Innovator | 80.19% | 51.58% | 11.5% |
|  | (2.87%) | (1.87%) | (0.98%) |
| Nominal Innovator | 9.83% | 31.55% | 36.27% |
|  | (2.01%) | (1.74%) | (1.45%) |
| Non-Innovator | 9.97% | 16.87% | 52.21% |
|  | (2.17%) | (1.27%) | (1.49%) |

*Source: 2014 Rural Establishment Innovation Survey.*

Table D provides information on the intersection of the innovation and design orientations in nonmetro establishments. The most critical row is the top one, which confirms that the rural share of design-integrated and design-last-finish establishments is lower than the national average. However, the differences are clearly in degree—the results do not support the presumption that systematic design is absent from rural economies. The intersection of design orientation and innovation orientation for nonmetro establishments resembles what is found in the nation as a whole: design-integrated establishments are overwhelmingly substantive innovators, while a plurality of design-last-finish establishments are also substantive innovators. Here, too, the off-diagonal cells (design-integrated non-innovators and substantive innovators with no systematic design approach) are not empty.

**Table D: Innovation Orientation by Design Orientation for Nonmetropolitan Establishments**

|  | Design Integrated | Design Last Finish | No Systematic Design |
| --- | --- | --- | --- |
| Percent of All Establishments | 4.76% (0.64%) | 28.28% (1.01%) | 66.97% (1.08%) |
| Substantive Innovator | 83.49% (5.92%) | 41.31% (1.63%) | 9.07% (0.77%) |
| Nominal Innovator | 11.04% (4.16%) | 38.09% (1.59%) | 41.00% (2.49%) |
| Non-Innovator | 5.47% (2.16%) | 20.53% (1.35%) | 49.93% (2.38%) |

*Source: 2014 Rural Establishment Innovation Survey.*

The identification of different design orientations in rural establishments addresses questions of existence and commitment, when it comes to design, but does design matter for these organizations? Clearly, if a strong commitment to design were associated with employment or wage growth, with the penetration of export markets, or with firm survivability, then promoting rural design might provide observable development benefits. Unfortunately, the data needed to test for the impact of design will not be available for several more years--when performance data for establishments in the dataset may be linked to administrative data. Linking the REIS dataset to the Longitudinal Business Database, along with other restricted use establishment microdata available through the Census Bureau’s Research Data Centers, is currently being investigated. Meanwhile, retrospective performance data reported by respondents may provide some hints on the impacts we may expect to see.

REIS asks a number of questions about establishment performance in the year, or past 3 years, prior to the survey. The average responses by design-orientation class are provided in Table E. In 2013, design-integrated establishments reported a significantly higher share of sales from new or improved products than did design-last-finish establishments. Establishments with no systematic approach to design reported a significantly lower share of sales from new or improved products. Qualitative questions ask whether the establishment experienced an improvement in various performance metrics over the past 3 years, and design-integrated establishments were most likely to respond affirmatively. Particularly notable is the much larger share of establishments with a systematic approach to design that began exporting, relative to establishments with no approach to design.

**Table E: Self-reported performance indicators by design orientation, Nonmetropolitan Establishments**

|  | Design Integrated | Design Last Finish | No Systematic Design |
| --- | --- | --- | --- |
| % of 2013 sales from new or improved products | 21.72* | 16.82* | 9.26 |
|  | (1.65) | (0.787) | (0.498) |
| In the past 3 years, the establishment: |  |  |  |
| Increased the variety of goods or services offered | 90.15%*  (1.71%) | 78.15%*  (1.11) | 50.29%  (1.02%) |
| Increased market share or entered new markets | 85.46%*  (2.47%) | 69.86%*  (1.19%) | 42.31%  (1.02%) |
| Began exporting goods or services | 37.52%*  (3.09%) | 15.27%*  (0.88%) | 3.76%  (0.30%) |
| Reduced time to respond to customer needs | 69.94%  (3.01%) | 61.24%*  (1.29%) | 39.76%  (0.99%) |
| Improved flexibility of production or service provision | 80.24%*  (2.63%) | 71.38%*  (1.20%) | 36.83%  (0.96%) |
| Increased capacity of production or service provision | 82.17%*  (2.25%) | 68.24%*  (1.26%) | 37.62%  (0.96%) |
| Reduced labor costs per unit output | 56.62%  (3.34%) | 42.13%*  (1.29%) | 21.49%  (0.81%) |
| Reduced materials and energy required per unit output | 49.89%*  (3.37%) | 34.38%*  (1.23%) | 13.74%  (0.65%) |
| Improved worker satisfaction or reduced worker turnover | 65.52%  (3.32%) | 69.26%*  (1.59%) | 48.56%  (1.02%) |

*Source: 2014 Rural Establishment Innovation Survey.*

** indicates estimate is statistically different from estimate to right at 5% level.*

The preliminary findings suggest that design orientation may matter for the economic outcomes that rural residents care about. While definitive findings will be possible when objective performance data become available, these initial results warrant a closer look at the “rural design” phenomenon.

External validation of the innovation measure developed in REIS has been able to go farther than is possible with the design measure. The auxiliary variables for the innovation LCA do not include patents or R&D activities or expenditures, which are widely regarded as proxies for innovation. Comparing the ranking of industries in REIS by the percentage of establishments classified as substantive innovators with the ranking of industries by the National Science Foundation with respect to patents and R&D expenditures could provide evidence that the REIS innovation measure is capturing the innovation phenomenon. Clearly, if the rankings are not correlated then the value of the measure would be doubtful. Unfortunately, the same type of external validation is not available for the design measure. Because design patents are included in the design-ladder LCA, a strong positive correlation in REIS and design patent rankings that could be constructed from U.S. Patent and Trademark Office data is nearly assured.

Results from a rank order comparison using the 2-digit NAICS industry classification is provided in Table F. With respect to patent applications the REIS rank order is nearly identical with the exception of Information (NAICS 51) swapping places with Manufacturing (NAICS 31-33). The rank order correlation is lowest for R&D expenditures per establishment with Mining (NAICS 21) climbing to third spot but being ranked last in REIS.

**Table F: External Validation of Latent Class Structure Using NSF 2-digit NAICS Rankings**

|  |  | National Science Foundation | | |  | REIS | |
| --- | --- | --- | --- | --- | --- | --- | --- |
| NAICS | 2-digit NAICS category | Rank of Patent Applications Per Establishment | Rank of Patents Issued Per Establishment | Rank of R&D Expenditures Per Establishment |  | Percent of Establishments in Substantive Innovator Class | Rank |
|  |  |  |  |  |  |  |  |
| 31–33 | Manufacturing | 1 | 1 | 1 |  | 34.21 | 2 |
| 51 | Information | 2 | 2 | 2 |  | 36.28 | 1 |
| 54 | Professional/scientific/technical services | 3 | 4 | 4 |  | 29.98 | 3 |
| 42 | Wholesale trade | 4 | 3 | 6 |  | 28.96 | 4 |
| 48–71 | Non-manufacturing industries | 5 | 6 | 5 |  | 26.17 | 5 |
| 21 | Mining | 6 | 5 | 3 |  | 16.79 | 6 |
|  | Rank Order Correlation with REIS Rank | 0.943 | 0.829 | 0.543 |  |  |  |

*Source: Shackelford (2013) and 2014 Rural Establishment Innovation Survey*

A tougher test is to compare the rank orders from the more disaggregate 4-digit NAICS industry classification that is provided in Table G. Only the most highly ranked industries are provided at the 4-digit level, or as a composite of 2 or 3 4-digit industries. Industries ranking much lower are combined in 2-digit or a composite of 2-digit industries. Here again, the top two industries in the NSF patents application rank swap places with the top two REIS industries. However, the much smaller cell size for the REIS comparison makes its rank order subject to greater sampling error. For example, Computer and electronic products: Communications equipment (NAICS 3342) is ranked last in REIS but is no lower than 7^th^ in any of the NSF ranks. The low rank in REIS is partly explained by no substantive innovators being identified in metro counties—observations with much larger sample weights given the oversampling of rural establishments. The rank order correlation between patent applications and REIS substantive innovator share rises to 0.433 when NAICS 3342 is removed.

**Table G: External Validation of Latent Class Structure Using NSF 4-digit NAICS Rankings**

NSF REIS

| NAICS | 4-digit NAICS category | Rank of Patent Applications Per Establish-ment | Rank of Patents Issued Per Establish-ment | Rank of R&D Expenditures Per Establish-ment | Percent of Establish-ments in Substantive Innovator Class | Rank |
| --- | --- | --- | --- | --- | --- | --- |
| 3254 | Chemicals: Pharmaceuticals and medicines | 1 | 4 | 1 | 52.72 | 2 |
| 3345 | Computer and electronic products: Navigational/measuring/electromedical/control instruments | 2 | 7 | 8 | 54.41 | 1 |
| 3364 | Aerospace products and parts: All | 3 | 5 | 3 | 31.37 | 12 |
| 3344 | Computer and electronic products: Semiconductor and other electronic components | 4 | 1 | 4 | 42.43 | 6 |
| 3341, 3343, 3346 | Computer and electronic products: Computer equipment/other electronic products | 5 | 2 | 5 | 34.57 | 10 |
| 5112 | Information: Software publishers | 6 | 6 | 6 | 35.92 | 8 |
| 3342 | Computer and electronic products: Communications equipment | 7 | 3 | 2 | 6.43 | 18 |
| 3251 | Chemicals: Basic chemicals | 8 | 8 | 9 | 17.12 | 17 |
| 325_ | Chemicals: Other | 9 | 10 | 10 | 48.87 | 5 |
| 3391 | Medical equipment and supplies: All | 10 | 11 | 12 | 27.39 | 15 |
| 5417 | Professional/scientific/technical services: Scientific research and development services | 11 | 12 | 11 | 51.72 | 3 |
| 3361–3363 | Automobiles/bodies/trailers/parts: All | 12 | 9 | 7 | 34.87 | 9 |
| 31__-33__ | Manufacturing nec, other: All | 13 | 13 | 13 | 33.44 | 11 |
| 51__ | Information: Other information, other | 14 | 14 | 15 | 36.31 | 7 |
| 5415 | Professional/scientific/technical services: Computer systems design and related services | 15 | 15 | 14 | 51.06 | 4 |
| 5413 | Professional/scientific/technical services: Architectural/engineering/related services | 16 | 16 | 16 | 28.51 | 13 |
| 54__ | Professional/scientific/technical services: Other | 17 | 17 | 17 | 24.28 | 16 |
| 21-23 42–81 | Nonmanufacturing nec, other: All | 18 | 18 | 18 | 27.77 | 14 |
|  | Rank Order Correlation with REIS Rank | 0.348 | 0.164 | 0.195 |  |  |
|  | Rank Order Correlation with REIS Rank excluding 3342 | 0.433 | 0.326 | 0.389 |  |  |

*Source: Shackelford (2013) and 2014 Rural Establishment Innovation Survey*

**Table H: Associations between Local Estimates of Design Orientation and Employment and Wage Growth, and Change in Number of Establishments, 2010-2014 with Industry Controls Reported (Table 6)**

|  | Employment Growth | |  | Avg Weekly Wages | |  | Change in Establishments | | |
| --- | --- | --- | --- | --- | --- | --- | --- | --- | --- |
| Variable | Parameter | Pr > \|t\| |  | Parameter | Pr > \|t\| |  | Parameter | Pr > \|t\| |  |
|  | Estimate |  |  | Estimate |  |  | Estimate |  |  |
| Intercept | 283.02836  (148.6117) | 0.0570 |  | -114.103 (61.1749) | 0.0623 |  | 0.71054  (3.538) | 0.8409 |  |
| Pr. Design Integrated | 6.22278  (35.8675) | 0.8623 |  | **52.6855 (14.8087)** | **0.0004** |  | 0.30663  (0.8502) | 0.7184 |  |
| Pr. Design Last Finish | **53.45583**  **(27.48957)** | **0.0519** |  | -8.50351 (11.3185) | 0.4526 |  | 0.29557  (0.6446) | 0.6466 |  |
| Population | 0.00013886  (0.0002) | 0.4665 |  | -0.0003  (0.00008) | <.0001 |  | -0.00002  (0.000004) | 0.0003 |  |
| Natural Amenity Scale | 32.06436  (5.03843) | <.0001 |  | -1.47495  (2.0751) | 0.4773 |  | -0.01459  (0.11946) | 0.9028 |  |
| Median Contract Rent | -0.42491  (0.13118) | 0.0012 |  | 0.02535 (0.05406) | 0.6392 |  | 0.00403  (0.0031) | 0.1959 |  |
| Share of 25-44 yo College Grads | 79.53659  (195.5923) | 0.6843 |  | 56.89083 (80.518) | 0.4799 |  | 2.73749  (4.5707) | 0.5493 |  |
| ind313 | -134.31319 | 0.7272 |  | 102.04689 | 0.5196 |  | -1.11415 | 0.8491 |  |
| ind334 | -153.21640 | 0.3178 |  | 191.89314 | 0.0024 |  | -1.38945 | 0.7035 |  |
| ind326 | -46.41068 | 0.7518 |  | 190.71365 | 0.0016 |  | -1.87847 | 0.5910 |  |
| ind325 | -115.69319 | 0.4276 |  | 345.92607 | <.0001 |  | -0.40999 | 0.9060 |  |
| ind322 | -215.31995 | 0.1655 |  | 257.45925 | <.0001 |  | -1.35068 | 0.7149 |  |
| ind335 | -50.93009 | 0.7337 |  | 144.23834 | 0.0193 |  | -1.59677 | 0.6543 |  |
| ind339 | -19.43600 | 0.9003 |  | 195.94711 | 0.0022 |  | -1.36804 | 0.7111 |  |
| ind333 | -16.44381 | 0.9104 |  | 241.43360 | <.0001 |  | -1.89100 | 0.5867 |  |
| ind324 | -148.46723 | 0.4505 |  | 443.45013 | <.0001 |  | -0.27773 | 0.9527 |  |
| ind315 | -135.59318 | 0.5104 |  | 150.86377 | 0.0753 |  | -1.35280 | 0.7828 |  |
| ind336 | 239.84252 | 0.0963 |  | 181.80354 | 0.0022 |  | -1.91599 | 0.5769 |  |
| ind517 | -145.14633 | 0.3367 |  | 227.00929 | 0.0003 |  | -3.26782 | 0.3637 |  |
| ind311 | -126.03938 | 0.3761 |  | 205.85132 | 0.0005 |  | -1.32365 | 0.6951 |  |
| ind331 | -97.82608 | 0.5170 |  | 322.56363 | <.0001 |  | -1.16532 | 0.7459 |  |
| ind487 | -163.30325 | 0.4768 |  | 35.53981 | 0.7068 |  | -1.56032 | 0.7713 |  |
| ind425 | -174.20222 | 0.3796 |  | 347.74607 | <.0001 |  | 0.17725 | 0.9701 |  |
| ind312 | -88.12590 | 0.6641 |  | 92.60901 | 0.2676 |  | 1.68909 | 0.7259 |  |
| ind518 | -84.48015 | 0.9057 |  | 124.65401 | 0.6711 |  | -1.91401 | 0.9103 |  |
| ind332 | -34.25467 | 0.8174 |  | 230.89773 | 0.0002 |  | -1.80123 | 0.6102 |  |
| ind481 | -53.42028 | 0.9162 |  | 52.58932 | 0.8013 |  | -0.98567 | 0.9350 |  |
| ind314 | -201.51897 | 0.2846 |  | 105.43028 | 0.1738 |  | -2.23738 | 0.6180 |  |
| ind316 | -155.15964 | 0.8371 |  | 187.32539 | 0.5465 |  | 0.74835 | 0.9084 |  |
| ind486 | -167.63029 | 0.5488 |  | 431.93125 | 0.0002 |  | -3.70061 | 0.5784 |  |
| ind711 | -175.81417 | 0.3300 |  | 98.41652 | 0.1852 |  | -3.83989 | 0.3716 |  |
| ind712 | -170.33433 | 0.2972 |  | 151.61789 | 0.0242 |  | -1.34814 | 0.7285 |  |
| ind519 | -173.61353 | 0.4371 |  | 236.82940 | 0.0107 |  | -1.23227 | 0.8157 |  |
| ind511 | -136.65326 | 0.3540 |  | 176.08136 | 0.0037 |  | -0.96841 | 0.7826 |  |
| ind515 | -159.89671 | 0.3399 |  | 165.84579 | 0.0162 |  | -2.00949 | 0.6146 |  |
| ind551 | -58.65336 | 0.6807 |  | 310.84791 | <.0001 |  | 0.04261 | 0.9900 |  |
| ind323 | -192.44192 | 0.1954 |  | 150.14492 | 0.0142 |  | -2.81883 | 0.4256 |  |
| ind327 | -83.71170 | 0.5894 |  | 186.47979 | 0.0035 |  | -1.33854 | 0.7170 |  |
| ind424 | -150.76536 | 0.2918 |  | 207.90192 | 0.0004 |  | -2.67333 | 0.4326 |  |
| ind423 | -82.80891 | 0.5731 |  | 208.16122 | 0.0006 |  | -1.04365 | 0.7656 |  |
| ind337 | -55.29573 | 0.7182 |  | 156.78775 | 0.0130 |  | -3.60846 | 0.3196 |  |
| ind522 | -163.26706 | 0.2813 |  | 198.06129 | 0.0015 |  | -2.90498 | 0.4209 |  |
| ind321 | -107.21420 | 0.4608 |  | 233.63889 | <.0001 |  | -3.54322 | 0.3061 |  |
| ind541 | -120.68249 | 0.4054 |  | 219.24096 | 0.0002 |  | 1.94659 | 0.5731 |  |
| ind212 | 751.77674 | <.0001 |  | 444.23108 | <.0001 |  | -2.50749 | 0.4631 |  |
| ind524 | -122.83881 | 0.4842 |  | 188.98560 | 0.0090 |  | -0.41384 | 0.9212 |  |
| ind488 | -146.32780 | 0.3996 |  | 183.85175 | 0.0102 |  | -0.97686 | 0.8134 |  |
| ind213 | 1366.72385 | <.0001 |  | 429.63028 | <.0001 |  | 44.47013 | <.0001 |  |
| ind512 | -135.85482 | 0.5208 |  | 216.61180 | 0.0129 |  | -1.82670 | 0.7119 |  |
| ind484 | -42.02950 | 0.7717 |  | 218.34306 | 0.0003 |  | 0.99174 | 0.7738 |  |
| ind485 | -49.33006 | 0.7505 |  | 165.44595 | 0.0096 |  | -2.14816 | 0.5557 |  |
| ind523 | -153.22637 | 0.7091 |  | 594.85263 | 0.0004 |  | 0.70325 | 0.9427 |  |
| Adj R-Sq | 0.356 |  |  | 0.1911 |  |  | 0.199 |  |  |
| N | 2382 |  |  | 2380 |  |  | 2403 |  |  |

*Pr. No Systematic Design excluded category. Source: 2014 Rural Establishment Innovation Survey, 2009-2013 Pooled ACS, ERS Natural Amenity Scale, BLS Quarterly Census of Employment and Wages 2010 and 2014*

## References

1. Galindo-Rueda, F, Millot, V. Measuring design and its role in innovation. OECD Science, Technology and Industry Working Paper 2015/01. Paris: OECD Publications; 2015

2. Bessant, J, Caffyn, S, Gallagher, M. An evolutionary model of continuous improvement behavior. Technovation 2001 21: 67–77.

3. Wojan, T, Parker, T. Innovation in the rural nonfarm economy: its effect on job and earnings growth, 2010-2014. Economic Research Report 237. Washington, DC: Economic Research Service; 2017.

4. . McCutcheon, AL. Basic concepts and procedures in single- and multiple-group latent class analysis. In Hagenaars, JA, McCutcheon, AL, editors. Applied Latent Class Analysis. Cambridge: Cambridge University Press, 2002. p. 56-87.
